# Artificial Intelligence and the Evolving Paradigm of Lung Cancer Management

**DOI:** 10.3390/cancers18122012

**Published:** 2026-06-22

**Authors:** Russell Seth Martins, Yousif Hanna, Andrea L. Axtell

**Affiliations:** 1 Department of Surgery, University of Wisconsin School of Medicine and Public Health, 600 Highland Avenue, Madison, WI 53705, USA; rmartins@uwhealth.org; 2 Division of Cardiothoracic Surgery, Department of Surgery, University of Wisconsin School of Medicine and Public Health, 600 Highland Avenue, Madison, WI 53705, USA

**Keywords:** artificial intelligence, deep learning, machine learning, neural networks, robotic surgery, non-small cell lung cancer

## Abstract

Lung cancer remains the leading cause of cancer-related mortality worldwide, largely due to late-stage diagnosis, biological heterogeneity, and persistent challenges in staging and treatment selection. This narrative review summarizes current and emerging applications of AI across lung cancer screening and early detection, imaging-based staging and prognostication, tissue and liquid biopsy-based tumor characterization, treatment planning, surgical and intraoperative guidance, and drug discovery. In imaging, deep learning models have demonstrated high performance in pulmonary nodule detection, risk stratification, and prediction of molecular alterations, while also showing promise in improving screening efficiency and reducing interpretive variability. In pathology and liquid biopsy domains, AI enables prediction of driver mutations, immunotherapy response, and survival outcomes directly from histopathology slides, circulating tumor DNA, and other blood-based biomarkers, facilitating minimally invasive precision oncology approaches. In treatment planning and delivery, AI systems are being developed to support clinical decision-making, surgical planning (through advanced image segmentation and delineation of operative anatomy), and intraoperative navigation through robotic and computer vision-enabled platforms. Despite these advances, significant barriers remain, including limited real-world validation, algorithmic biases, workflow integration issues, and unresolved ethical and legal concerns. Future progress will depend on the development of transparent, clinically validated, and generalizable AI systems that augment rather than replace the expertise of clinical providers and healthcare teams. Active engagement from pulmonologists, oncologists, radiologists, and thoracic surgeons will be essential in guiding safe implementation and ensuring that AI-driven innovations translate into meaningful improvements in patient outcomes.

## Simple Summary

In this narrative review, we examine how artificial intelligence is being explored and applied across the continuum of lung cancer care. Key applications include the detection of lung nodules, stratifying risk associated with nodules, predicting treatment response, and understanding molecular changes driving disease processes. Artificial intelligence may also support clinical decision-making and planning for cancer operations. However, major barriers to the applications of artificial intelligence include limited real-world validation, algorithmic biases, workflow integration challenges, and complex ethicolegal issues. Active engagement from pulmonologists, oncologists, radiologists, and thoracic surgeons will be essential to ensure that AI-driven innovations translate into meaningful improvements in patient outcomes.

## 1. Introduction

Lung cancer continues to be one of the most common and deadly cancers worldwide, with 12% of new cancer cases and 19% of cancer-related deaths being attributable to lung cancer [1]. Non-small cell lung cancer (NSCLC) accounts for the majority of cases, with over 2 million new cases around the globe annually [1]. Despite advances in diagnosis and management over the past few decades, prognosis remains poor for patients diagnosed with NSCLC. The 5-year survival rate for patients with NSCLC in the United States (US) is estimated to be around 26–32% across all stages combined [2]. This poor overall prognosis is largely driven by late-stage presentation, when curative surgical resection is no longer feasible, as well as the biologic heterogeneity of the disease. In addition, limitations in early detection, challenges in accurate staging, and variable treatment response further contribute to suboptimal outcomes. These challenges underscore the need for more precise, efficient, and individualized approaches to the management of this disease.

In recent years, artificial intelligence (AI) has emerged as a powerful tool with the potential to reshape multiple aspects of lung cancer care. Briefly, AI refers to a broad set of computational techniques that enable machines to analyze complex data, identify patterns, generate predictions, and even execute interventions with minimal human intervention. In the context of lung cancer, the growing availability of large-scale datasets spanning clinical, radiological, and molecular domains has created an ideal environment for the application of AI-driven methods. These technologies offer the potential to augment human decision-making, improve consistency, and uncover insights that may not be readily apparent through traditional approaches.

The integration of AI into lung cancer care is particularly promising given the increasing ubiquitousness of multidisciplinary management approaches and longitudinal care [3]. As research and clinical adoption continue to expand, understanding the role of AI in this field is essential to realize its full potential for all stakeholders involved in delivering lung cancer care. In this narrative review, we explore the role of artificial intelligence across the continuum of lung cancer management, with a focus on its applications in detection, staging, treatment planning, and its emerging clinical implications. Rather than employing a formal systematic review methodology, the literature discussed was selected to provide representative coverage of key technological advances, clinical applications, implementation challenges, and future directions within this rapidly evolving field. It is also important to recognize that the studies discussed in this review span a spectrum of evidence maturity, ranging from retrospective model development studies to externally validated algorithms and early prospective evaluations. Accordingly, many AI applications remain investigational, and reported performance metrics should be interpreted within the context of the underlying study design, validation approach, and stage of clinical development.

## 2. Lung Cancer Screening and Early Detection Through Imaging

The most recent data suggests that less than 20% of eligible patients in the US receive lung cancer screening, with the number being less than 10% in some states [4]. A low-dose CT scan remains the cornerstone of lung cancer screening, and it has been repeatedly shown that adherence to screening guidelines leads to a stage shift in detection and resultant reduction in lung cancer mortality [5]. AI-driven tools are increasingly being developed to augment the detection, characterization, and management of pulmonary nodules. These technologies have the potential to improve diagnostic accuracy, optimize resource utilization, and ultimately enhance patient outcomes.

Automated detection of lung nodules on imaging represents one of the most mature applications of AI in lung cancer [6], with models often achieving sensitivities comparable to or exceeding those of radiologists in controlled settings. For example, the deep learning system developed by Ardila et al. demonstrated an area under the curve of 94% for lung cancer prediction using low-dose CT, outperforming radiologists in certain settings without prior imaging [7]. AI can also be used to augment reading of simple chest radiographs to detect incidental pulmonary nodules with malignant features [8]. AI models may also be able to improve the false positive rate in lung cancer screening. Historical data from the National Lung Screening Trial [9] showed that over 95% of nodules identified by radiologists were non-malignant, although subsequent development of the Fleischner [10] and Lung-RADS [11] criteria have led to improvements in the false positive rates. Contemporary AI models have been able to independently achieve low false positive rates based on imaging [12]. However, sensitivity and specificity are typically inversely related, and models may have trade-offs between one or the other, thus limiting their independent use in screening and detection and necessitating human oversight [13]. This trade-off underscores the importance of positioning AI as a decision-support tool rather than a fully autonomous diagnostic system at present. As interest and research into the applications of AI for lung cancer screening and detection continue to grow, national societies and governmental agencies such as the American College of Radiology and the National Cancer Institute are developing initiatives to support the development of AI models [14].

In early 2026, the US Food and Drug Administration (FDA) granted 510 (k) clearance for an AI-powered lung cancer screening (LCS) software for the detection and diagnosis of lung lesions suspicious for cancer [15]. In manufacturer performance testing on a lung cancer screening reference population, this software demonstrated 93.3% sensitivity, 92.4% specificity, and a 99.9% negative predictive value (NPV) [16]. The product is planned for a phased launch in the US, prioritizing regions with the highest lung cancer screening volumes [16]. Regulatory milestones such as this reflect the growing transition of AI tools from research settings into real-world clinical deployment. It is unclear what the long-term impact of AI models in lung cancer screening will be when they undergo mass clinical uptake, but they will initially function primarily as an augmentative tool to human radiologists [13]. The implications of AI integration for radiologist workload are complex. On one hand, AI has the potential to reduce workload by automating time-consuming tasks such as nodule detection, segmentation, and volumetric measurements [17,18]. On the other hand, AI also has the potential to increase adherence to lung cancer screening by improving the extraction and integration of clinical and social determinants of health from large electronic health record datasets [19,20]. In addition, early deployment phases of such systems may reveal workflow integration challenges, including the need for validation, quality assurance, and radiologist oversight of AI-generated outputs.

## 3. Lung Cancer Staging, Prognostication, and Tumor Characterization

Accurate staging and prognostication remain central to the management of lung cancer and directly inform treatment selection and expected outcomes. Traditionally, staging relies on a combination of radiologic imaging, histopathologic assessment, and clinical evaluation, primarily guided by the TNM classification system. However, AI is increasingly being explored as a means of enhancing the precision and predictive power of lung cancer staging and tumor characterization through multimodal data integration.

Even prior to the use of AI-based methods, conventional statistical analysis has been used to determine prognosticative information for lung cancer imaging [21]. These earlier approaches typically relied on predefined imaging features and manually selected variables, which inherently limited the depth and scalability of feature extraction. Thus, traditional statistical models are limited by the quality and quantity of information being extracted. AI amplifies this ability by being able to extract more granular information from CT and PET imaging than feasible or even possible for human radiologists. This shift from human-defined features to automatically learned representations enables the identification of subtle imaging patterns (such as fine textural variations, pixel-level intensity patterns, and minute spatial or temporal changes) that may not be visually appreciable by human radiologists but have crucial value as datapoints in lung cancer care. Previous studies have shown the ability of AI models to predict tumor histology [22], programmed death-ligand 1 (PD-L1) [23] and epidermal growth factor receptor (EGFR) [24] status, lymph node [25] and distant organ [26] metastasis, treatment response and adverse reactions to immunotherapy [27], post-treatment response to immunotherapy [28,29], overall survival after treatment [30,31], and local or distant recurrence [32,33]. Collectively, these advances emphasize the growing role of imaging as a high-dimensional biomarker that can support more individualized and data-driven oncologic decision-making.

Another key application of AI in modern lung cancer management is in endobronchial systems used in tissue sampling. Robotic bronchoscopy systems improve lesion localization and navigation during biopsy procedures, particularly for small or peripherally located nodules that are traditionally difficult to access [34]. These systems leverage a combination of pre-procedural CT imaging, real-time navigation, and AI-enhanced segmentation to create dynamic, three-dimensional airway maps that guide surgeons or interventional pulmonologists to target lesions with greater precision. AI also assists these systems in overcoming long-standing shortcomings of CT-to-body divergence by using computer vision and shape-sensing technology to dynamically compare real-time anatomy with pre-procedure imaging and adjust the navigation pathway accordingly [35]. Reportedly, AI-powered navigation can boost the real-time intra-procedural processing power of some endobronchial systems by 260%, enabling the execution of complex algorithms that enhance navigation accuracy and procedural confidence [36]. AI has also been explored in endobronchial ultrasound (EBUS) for lymph node assessment and guidance of transbronchial needle aspiration (TBNA), with preliminary studies suggesting possible improvements in diagnostic yield [37,38,39,40,41]. As these technologies evolve, they hold promise not only for improving diagnostic efficiency but also for enabling earlier and more precise tissue acquisition, which is critical in the era of molecular profiling and personalized lung cancer therapy.

Furthermore, AI is also being applied to blood-based molecular assays, including circulating tumor DNA (ctDNA), cell-free DNA (cfDNA), circulating tumor cell (CTC), and extracellular vesicle (EV) analysis. These granular and complex data points often exceed the ability of traditional statistical models to draw inferences. In contrast, AI models are uniquely suited to synthesize vast amounts of granular, multi-dimensional data, and can detect subtle yet complex signals and relationships that may inform clinical decision-making [42,43,44]. Machine learning applied to cell-free DNA (cfDNA) methylation patterns has demonstrated the ability to detect lung cancer even at very low DNA concentrations [45]. In addition, cfDNA-based models using random forest algorithms can identify EGFR mutation status and support early detection through transcription start site signal analysis [46]. AI-based EV profiling has shown utility in distinguishing lung cancer from other malignancies [47,48]. Prognostic modeling using AI algorithmic analysis of ctDNA profiles has also been effective in predicting outcomes in patients with non-small cell lung cancer following chemoradiation [49]. Other applications of AI in the liquid biopsy sphere center around lung cancer detection, which can be a powerful adjunct to AI-powered radiomics discussed earlier in this review. AI models trained on circulating non-coding RNAs have achieved high diagnostic performance for lung cancer detection, with reported sensitivity of 94% and specificity of 87% using a simple blood-based assay [50]. cfDNA fragmentation analysis has demonstrated 94% sensitivity and 80% specificity and has been shown to reduce false-positive rates by approximately 50% compared with standard low-dose CT screening [51]. In addition, cfDNA methylation-based risk stratification models have shown promise in refining lung cancer risk classification among individuals undergoing low-dose CT screening [52]. Several of these AI-powered liquid biopsy solutions for risk stratification, early detection of tumors and actionable mutations, and treatment monitoring are currently on the market and are increasingly becoming a part of routine clinical workflows at many institutions. As these technologies mature, they hold the potential to complement imaging- and solid tissue-based assessment and contribute to a more comprehensive, minimally invasive approach to lung cancer surveillance and management.

Finally, AI-based pathology analysis is becoming an important tool in tumor characterization, due to its ability to predict genetic mutations and clinical outcomes directly from histological slides. AI enables extraction of quantitative morphologic patterns that extend far beyond conventional human visual assessment. AI models have demonstrated feasibility in predicting key lung cancer driver mutations directly from histopathology images of NSCLC, such as PD-L1, EGFR, and Kirsten rat sarcoma viral oncogene homolog (KRAS), amongst others [53,54,55,56,57,58]. This allows for the generation of virtual molecular profiles directly from routine pathology specimens, potentially providing an alternative to extensive molecular sequencing. In particular, AI models equipped to detect multi-molecular proteomic panels from standard hematoxylin and eosin (H&E) slides are able to improve prognostic accuracy by 22% and immunotherapy response prediction by up to 39% compared with conventional clinicopathological and molecular biomarkers [57]. As these models become commercially available and gain widespread adoption, they are set to transform the field of histopathology in lung cancer by enabling more information extraction from digital slides than ever before.

## 4. Treatment Planning and Delivery

In the setting of treatment planning, AI may function not as an autonomous decision-maker but as an augmentation tool that synthesizes large, multimodal datasets to support more precise and individualized treatment strategies. This includes integrating radiologic, pathologic, molecular, and clinical data streams into unified predictive frameworks that can be updated dynamically as new patient information becomes available. As lung cancer management becomes increasingly complex and multidisciplinary, these systems offer the potential to reduce variability in care, streamline decision-making, and improve both surgical and oncologic outcomes.

AI-guided clinical decision-support systems (CDSS) are envisioned to assist clinicians in selecting optimal therapeutic pathways by integrating individual patient-level variables such as demographics, tumor stage, radiologic features, molecular biomarkers, and comorbidities. Unlike static clinical guidelines and pathways, these models can in theory continuously refine predictions as new data are incorporated, allowing for more adaptive and data-driven treatment planning. However, there is much work to be done before AI clinical decision support systems are able to be implemented in clinical practice. For example, IBM’s Watson for Oncology was initially developed to assist oncologists by rapidly synthesizing literature and recommending evidence-based cancer treatments. Despite early enthusiasm and substantial investment, the system ultimately struggled with limited and non-representative training data, poor adaptability to local clinical guidelines, and inconsistent or even unsafe recommendations in real-world settings [59]. In recent times, large language models (LLMs) such as GPT-4 (OpenAI) and Claude Opus (Anthropic) have also been explored as CDSSs for lung cancer management, but this approach has also shown to lead to inaccurate or even outright harmful recommendations [60].

In the surgical management of lung cancer, AI is increasingly being applied to preoperative planning by improving anatomic delineation and functional prediction. For instance, deep learning segmentation algorithms applied to CT scans can generate 3D reconstructions of tumor boundaries and segmental pulmonary anatomy [61,62,63,64]. These reconstructions help surgeons determine whether a lesion is amenable to segmentectomy versus lobectomy by mapping tumor proximity to segmental bronchi and vessels [61,62,63,64]. Moreover, these models are also useful in identifying any aberrant anatomy, assisting with intraoperative parenchymal margins, and increasing surgeons’ perception of greater safety during the operation [61]. AI-based models have also been used to predict feasibility of lung-sparing resections in early-stage NSCLC by analyzing tumor location relative to anatomical segments and calculating residual functional lung volume after resection [61,62,63,64]. Machine learning models combining CT-based quantitative COPD scoring with spirometry data have been shown to accurately estimate postoperative FEV1 [65] and risk of pulmonary complications [66], providing data that is particularly useful in borderline surgical candidates with COPD.

Intraoperative AI integration with robotic systems represents another rapidly evolving field of applications. Some robotic surgery platforms are exploring computer vision modules that can identify anatomical landmarks and assist with dissection guidance. For example, augmented reality overlays can project tumor margins and vascular structures onto the surgical field, improving orientation and safety during lung operations [67]. Additionally, experimental systems using real-time optical flow analysis can track instrument motion and provide feedback on deviation from planned trajectories [68].

Finally, AI is also playing a central role in advancing personalized medicine and accelerating drug discovery [69]. Gene co-expression network analysis combined with survival data has been used to identify key hub genes involved in lung adenocarcinoma, drawing attention to potential therapeutic targets for drug development [70]. AI-integrated high-throughput screening approaches have also been used to identify existing FDA-approved drugs which may have roles in the treatment of non-small cell lung cancer [71]. Interestingly, AI-discovered drugs have a higher success rate in Phase I clinical trials than traditionally identified drugs (80–90% compared to 40–65%) [71]. These findings collectively suggest that AI may be capable of bridging discovery biology and translational medicine, shortening the pathway to clinically actionable treatments.

Table 1 summarizes the above discussion on the applications of AI across the spectrum of lung cancer care.

## 5. Ethicolegal Challenges

As AI becomes increasingly ubiquitous in healthcare, there are important ethical and legal considerations that must be addressed to ensure safe, equitable, and responsible use. Amongst these are issues related to data privacy and governance, algorithmic bias and fairness, transparency, regulatory oversight, and liability. These domains are interconnected, and shortcomings in one area often exacerbate challenges in others, underlining the need for a comprehensive and coordinated approach towards development of regulatory frameworks.

Data privacy is a primary concern, as AI systems rely on large volumes of patient data for training and validation. Ensuring the secure storage, transmission, and use of sensitive health information is essential to maintaining patient trust and complying with regulatory standards. While standard de-identification and data anonymization strategies are commonly employed, legitimate concerns remain regarding the potential for re-identification by increasingly sophisticated AI models, particularly when datasets are combined across sources [2,72,73,74]. In lung cancer, these concerns have been brought to light across studies integrating datasets of medical images, genomic data, and clinical records [75]. As data-sharing collaborations expand across institutions and even borders, the complexity of safeguarding patient information will continue to increase. It is crucial that existing national and international regulatory frameworks around the world be expanded to protect health data privacy across the growing technological and corporate stakeholders involved in healthcare-related AI.

Algorithmic bias represents another significant challenge. AI models are only as robust as the data on which they are trained, and it is well known that the underrepresentation of certain populations or characteristics can lead to biases and disparities in performance of AI models [76]. For example, several prominent cancer pathology databases are disproportionately composed of Caucasian patients, with minority groups collectively representing only a small fraction of included cases [77]. This lack of representation can introduce systematic bias into AI-based diagnostic models, as these systems often learn and reproduce patterns embedded within their training datasets [78]. Moreover, it has been demonstrated that pathology images may include features that are associated with patient race [79], increasing the risk of models relying on confounding signals rather than true disease-related pathological features on imaging. These limitations can ultimately lead to uneven diagnostic performance across populations and reinforce disparities in clinical outcomes. In lung cancer screening, standard criteria have historically resulted in fewer Black individuals at risk of lung cancer identified as being eligible for screening, compared to their White counterparts [80]. The performance of AI models in lung cancer radiomics has been shown to vary across different age groups and even amongst the histological subtypes of lung cancer [81]. Importantly, such biases are often not immediately apparent without deliberate and structured evaluation. Thus, comprehensive and systematic evaluation and auditing of bias in AI systems should become an essential step prior to any real-world implementation. Ongoing post-deployment monitoring and periodic recalibration may be necessary to identify performance drift and ensure equitable outcomes as patient populations and clinical practices evolve over time.

Transparency and explainability are critical for fostering clinician and patient trust. Many AI systems function as “black boxes,” making it difficult to understand how specific decisions are generated. As captains of the ship, physicians must be able to clearly communicate the rationale behind diagnostic and therapeutic decisions, particularly when these are informed by AI, in order to support shared decision-making and maintain patient trust. Equally important, physicians need to understand the reasoning underlying AI-generated outputs so they can critically evaluate, contextualize, and, when necessary, override recommendations that may not align with the clinical picture. A lack of explainability risks eroding clinician autonomy, undermining informed consent, and introducing uncertainty into high-stakes clinical discussions. In the context of insurance decision-making, limited transparency in AI-driven models is particularly concerning, as coverage determinations directly affect patient access to care and financial burden. With an increasing reliance on complex algorithms without clearly explainable criteria, there may be new challenges for patients and clinicians to understand, contest, or meaningfully appeal denials that may significantly impact treatment pathways [76]. Enhancing interpretability is therefore not only a technical goal but also a moral and legal imperative in maintaining accountability.

Lastly, the legal framework governing accountability for AI-related errors in lung cancer management remains uncertain, as it is unclear which liability standards should apply to these technologies. Determining responsibility in cases where AI contributes to diagnostic or therapeutic errors is not straightforward, particularly when decisions involve both human and machine input. Clear guidelines are needed to delineate the roles and responsibilities of clinicians, developers, and healthcare institutions. Some considerations involve holding manufacturers responsible for defects without requiring proof of fault [82]. However, these approaches are complicated by the adaptive nature of many AI systems, which continue to evolve after deployment as they are exposed to new data. The opposite end of the spectrum involves placing greater responsibility on clinicians or healthcare institutions, arguing that AI tools function as decision-support systems rather than autonomous agents, and therefore ultimate accountability should rest with the human operator who integrates these outputs into clinical care. Under this view, liability may arise from failure to appropriately interpret, override, or validate AI-generated recommendations in the context of established standards of care. Understandably, this may potentially be a source of significant cognitive distress for healthcare providers given the potential liability for decisions made using “black box” AI models that are not fully explainable. However, as AI becomes increasingly embedded in clinical workflows, healthcare institutions and providers may in the future face greater scrutiny not for using AI, but for failing to utilize available AI tools that are considered standard of care. As regulatory efforts advance and jurisdictions explore frameworks for high-risk AI systems, clearer guidance is needed to define responsibility among AI developers, healthcare institutions, and clinicians. Today, agencies across countries and globally are working to establish frameworks for the evaluation, approval, and monitoring of AI-based medical tools. These frameworks must balance the need for rigorous validation with the flexibility to accommodate iterative improvements, and regulatory oversight must continually evolve to keep pace with inevitable technological advancements in AI. Table 2 summarizes the above discussion ethicolegal implications of AI lung cancer care.

## 6. Conclusions

Lung cancer remains a leading cause of cancer-related mortality worldwide. In just a few short years, AI has already started to deliver immense impact across the full continuum of lung cancer care, including screening and early detection, imaging-based staging and prognostication, tissue and liquid biopsy-based tumor characterization, surgical planning and intraoperative guidance, systemic therapy optimization, and drug discovery. Across these domains, AI demonstrates the ability to extract high-dimensional information from diverse datasets, enabling more precise risk stratification, earlier detection of disease, and increasingly personalized therapeutic strategies. In parallel, the transition of multiple AI tools from experimental models to regulatory clearance underscores their growing clinical relevance and eventual ubiquitous presence in lung cancer care.

Despite these advances, significant limitations remain. Firstly, while statistical measures such as are important indicators of model performance, the ultimate value of AI systems will hinge on their ability to improve clinically meaningful outcomes, enhance workflow efficiency, support decision-making, and demonstrate benefit in real-world clinical practice. Given that relatively few AI models have reached widespread clinical implementation, published reports of real-world failures and implementation challenges remain limited. As adoption expands, these experiences will provide important insights into the safe and effective integration of AI into clinical practice. Future progress will depend on the development of transparent, clinically validated, and generalizable AI systems that augment rather than replace physician expertise. Oncologists and thoracic surgeons will play a central role in this evolution, not only as end-users, but as active collaborators in model development, validation, and oversight. Their domain expertise will be critical in ensuring that AI tools are clinically meaningful and aligned with patient-centered care. Ultimately, the most effective integration of AI into lung cancer management will emerge from a partnership between technological innovation and clinical judgment, with the shared goal of improving outcomes for patients.

## Figures and Tables

**Table 1:** Applications of artificial intelligence across the continuum of lung cancer care.

Domains	Potential Applications
Screening and Early Detection (Imaging)	Nodule detection and malignancy prediction on low-dose CT scan. Malignant nodule detection on chest radiographs Screening eligibility & adherence from EHR
Staging, Prognostication and Characterization (Imaging)	Predicting tumor histology Predicting PD-L1 and EGFR status Predicting lymph node & distant metastasis Predicting immunotherapy response & pneumonitis Predicting overall survival; local/distant recurrence
Tissue Sampling	Robotic bronchoscopy AI-assisted EBUS-TBNA lymph node assessment
Liquid Biopsy	Lung cancer screening and detection. Predicting clinical outcomes Determining treatment response Monitoring for recurrence
Pathology	Predicting driver mutations Predicting treatment response
Treatment Planning and Delivery	Clinical decision support 3D segmentation for resection planning Postoperative FEV1 & pulmonary complication risk Intraoperative augmented reality overlays & instrument tracking
Drug Discovery	Target & hub-gene identification; drug repurposing

**Table 2:** Ethicolegal challenges associated with artificial intelligence in lung cancer care and potential mitigation strategies.

Ethicolegal Issue	Relevance to Lung Cancer Care	Strategies for Mitigation
Data Privacy and Confidentiality	AI systems require large datasets across institutions, increasing the risk of patient re-identification despite anonymization.	Strengthen data governance policies Employ advanced de-identification techniques Utilize federated learning and privacy-preserving AI methods Expand national and international regulations governing health data sharing and AI development.
Data Ownership and Consent	Patients may be unaware that their data are being used to train AI systems	Develop transparent consent processes Establish clear policies regarding data ownership and secondary data use Incorporate dynamic consent models allowing patients greater control over data utilization.
Algorithmic Bias and Health Equity	Underrepresentation may result in unequal diagnostic accuracy, risk prediction, and treatment recommendations.	Ensure diverse and representative training datasets; perform subgroup analyses during model validation Implement mandatory bias audits and fairness assessments before and after deployment.
Generalizability and External Validity	Models trained in specific institutions or populations may perform poorly when applied to others	Require multicenter external validation Evaluate performance across demographic and geographic subgroups Establish minimum standards for real-world validation before implementation.
Transparency and Explainability	AI systems may generate recommendations without providing understandable reasoning	Promote development of explainable AI models Require documentation of model architecture, training data characteristics, and decision-making processes Integrate interpretable outputs into clinical workflows.
Clinician Autonomy and Oversight	Excessive reliance on AI may reduce independent clinical judgment or create automation bias.	Maintain human-in-the-loop decision-making Define AI as a decision-support rather than decision-making tool Provide clinician training on appropriate AI interpretation and limitations.
Informed Consent and Shared Decision-Making	Patients may not understand when AI contributes to healthcare decisions, potentially affecting informed consent processes.	Disclose AI involvement when clinically relevant Educate patients regarding the role and limitations of AI-assisted recommendations Develop standardized communication frameworks.
Insurance and Coverage Decisions	AI-assisted insurance authorization systems may lack transparency and potentially introduce bias into coverage determinations.	Require explainability of AI-assisted coverage decisions Establish mechanisms for appeals and human review Mandate regulatory oversight of payer AI systems.
Liability for AI-related Errors	Uncertainty exists regarding responsibility when AI contributes to healthcare-related errors or adverse outcomes	Establish clear legal frameworks defining responsibilities of clinicians, healthcare institutions, and AI developers Develop shared accountability models Clarify standards of care involving AI-assisted decision-making.
Adaptive Algorithms	AI systems may evolve after deployment, resulting in model drift or performance changes.	Implement continuous performance monitoring Require periodic revalidation and regulatory review Establish post-market surveillance systems for AI tools.
Regulatory Oversight and Approval Pathways	Existing regulatory frameworks may struggle to accommodate rapidly evolving AI technologies and iterative model updates.	Develop lifecycle-based regulatory approaches that include premarket evaluation, post-market surveillance, and update governance
Trust and Public Acceptance	Concerns regarding privacy, bias, explainability, and accountability may undermine patient and clinician confidence in AI-assisted care.	Foster transparency, stakeholder engagement, independent auditing, and publication of real-world performance data Emphasize patient-centered governance strategies.

## Data Availability

No new data were created or analyzed in this study. Data sharing is not applicable to this article.

## Abbreviations

The following abbreviations are used in this manuscript:

AI	Artificial Intelligence
CDSS	Clinical Decision Support System
cfDNA	Cell-free Deoxyribonucleic Acid
COPD	Chronic Obstructive Pulmonary Disease
CTC	Circulating Tumor Cell
CT	Computed Tomography
DNA	Deoxyribonucleic Acid
EBUS	Endobronchial Ultrasound
EGFR	Epidermal Growth Factor Receptor
EV	Extracellular Vesicle
FDA	Food and Drug Administration
FEV_1_	Forced Expiratory Volume in 1 Second
GPT-4	Generative Pre-trained Transformer 4
H&E	Hematoxylin and Eosin
IBM	International Business Machines
KRAS	Kirsten Rat Sarcoma Viral Oncogene Homolog
LLM	Large Language Model
Lung-RADS	Lung Imaging Reporting and Data System
NPV	Negative Predictive Value
NSCLC	Non-Small Cell Lung Cancer
PD-L1	Programmed Death-Ligand 1
PET	Positron Emission Tomography
TBNA	Transbronchial Needle Aspiration
TNM	Tumor, Node, Metastasis (staging system)
